# Perspectives on artificial intelligence-generated chronic disease risk predictions in early breast cancer: an international survey among radiation oncologists

**DOI:** 10.2340/1651-226X.2026.45901

**Published:** 2026-06-18

**Authors:** Frederik Voigt Carstensen, Belinda Bøgh Irankunda, Eva Batista, Desiree van den Bongard, Tanja Spanic, Sofie A. M. Gernaat, Helena Verkooijen, Maja Vestmø Maraldo

**Affiliations:** a Department of Oncology, Copenhagen University Hospital Rigshospitalet, Copenhagen, Denmark; b Breast Unit, Champalimaud Foundation, Lisbon, Portugal; c Department of Radiation Oncology, Amsterdam UMC, Cancer Center Amsterdam, Amsterdam, Netherlands; d Cancer Treatment and Quality of Life/Cancer Biology and Immunology, Amsterdam, Netherlands; e Europa Donna Slovenja, Ljubljana, Slovenia; f Division of Imaging and Oncology, University Medical Center Utrecht, Utrecht, Netherlands; g Department of Oncology, Zealand University Hospital Næstved, Næstved, Denmark; h Department of Clinical Medicine, University of Copenhagen, Copenhagen, Denmark

**Keywords:** Breast cancer, radiotherapy, artificial intelligence, risk prediction, chronic disease

## Introduction

Advances in breast cancer treatment have led to improved survival rates, with 83% of patients diagnosed in Europe surviving at least 5 years following diagnosis [1]. Early breast cancer (eBC) survivors have an elevated risk of developing chronic diseases such as cardiovascular disease (CVD), osteoporosis, lung disease and obesity [2–4], which can partly be explained by side effects of chemotherapy, antibody-treatment, and endocrine therapies [5–8]. Furthermore, unfavorable body composition and detrimental lifestyle is associated with increased risk of breast cancer recurrence [9, 10]. Recent studies have highlighted the long-term burden of treatment-related morbidity among breast cancer survivors, particularly CVD that may occur years after treatment completion. In addition, CVD risk varies according to treatment exposures and patient characteristics, underlining the need for improved risk assessment strategies in survivorship care [7, 8]. Consequently, strategies to enable earlier identification and prevention of chronic diseases are increasingly important.

Postoperative radiotherapy is a standard adjuvant treatment for most eBC patients with all patients referred for radiotherapy receiving a planning computed tomography (pCT)-scan. Recent developments in artificial intelligence (AI) have enabled extraction of clinically relevant imaging biomarkers from pCT-scans. Gal et al. showed that quantification of coronary artery calcium from pCT-scans is associated with future CVD risk in large eBC cohorts [11]. Furthermore, pCT-scans contain information about bone density, distribution of muscle- and fatty-tissue, and airway configuration, which may be used to predict the risk of developing osteoporosis, chronic obstructive pulmonary disease (COPD) and unfavorable body composition through AI-models [12–14].

Recently, we demonstrated that the majority of eBC patients were interested in receiving AI-generated predictions of their risk of developing chronic diseases, suggesting patient acceptance of such tools in oncology care and follow-up [15]. However, a successful implementation of AI-generated risk predictions in clinical practice also depends on the acceptance of the healthcare professionals, as they play a vital role in integrating, interpreting, and communicating such risk predictions to patients [16].

The aim of this study was to investigate radiation oncologists’ (ROs) attitudes toward AI-generated risk predictions of chronic diseases in patients with eBC referred for postoperative radiotherapy.

## Material and methods

The study was designed as a cross-sectional survey. The target population was European ROs treating patients with breast cancer.

An online survey was developed in REDCap. The survey consisted of a cover page with study information, and five pages with survey items. The survey included demographic items (age, country, location of institution) and eight questions: four questions assessing the perceived relevance for the eBC population of an AI-generated risk prediction of developing CVD, osteoporosis, COPD and unfavorable body composition, and four questions assessing the perceived usefulness in daily practice of such AI-models. The response scale was self-constructed with response options ‘*Yes*’, ‘*No*’, and ‘*Don’t know*’. Each question was accompanied by a decision support chart developed for a future decision impact trial.

The survey was approved by the ESTRO Scientific Council for distribution. The survey was promoted at two separate events at the ESTRO 2025 conference (May 3rd, 2025, and May 6th, 2025). On May 20th, 2025, the survey was distributed by ESTRO via e-mail to ROs with ESTRO membership and a self-proclaimed interest in breast cancer care. Access to the survey was provided via a direct link or QR-code.

### Data analysis

Responders were analyzed using descriptive statistics and grouped by European regions in accordance with UN Geoscheme [17]. Exploratory multivariate logistic regression was conducted. The variables included in the multivariate logistic regression were age (continuous), region (nominal categorical), and attitude toward AI-generated risk prediction, categorized as positive (‘*Yes*’) versus non-positive (‘*No*’ and ‘*Don’t know*’) (binary). Only survey questions with at least 10 non-positive responses were included in the regression analysis to reduce model instability. All analyses were performed using ‘R for Windows’ version 4.5.0 and ‘R Studio’ version 2024.12.1.

## Results

Data were collected from May 2025 to September 2025. The survey was accessed by 130 individuals, and 96 provided complete or partial responses. Median age was 47 years (range: 24–67). Responders represented 22 European countries (83.3%), as well as non-European regions (16.7%), with 92.7% affiliated with urban institutions (Table 1).

**Table 1:** Demographic information.

No. of fully or partially completed surveys	96
Median age, years	47
Age range, years	24–67
Country of practice (by European region^a^)	*n*
Eastern Europe	11 (11.5%)
Western Europe	29 (30.2%)
Northern Europe	11 (11.5%)
Southern Europe	29 (30.2%)
Non-European	16 (16.7%)
Location of institution	*n*
Urban	89 (92.7%)
Suburban	3 (3.1%)
Rural	4 (4.2%)
Remote	0 (0%)

a In accordance with UN Geoscheme.

**Figure 1:**
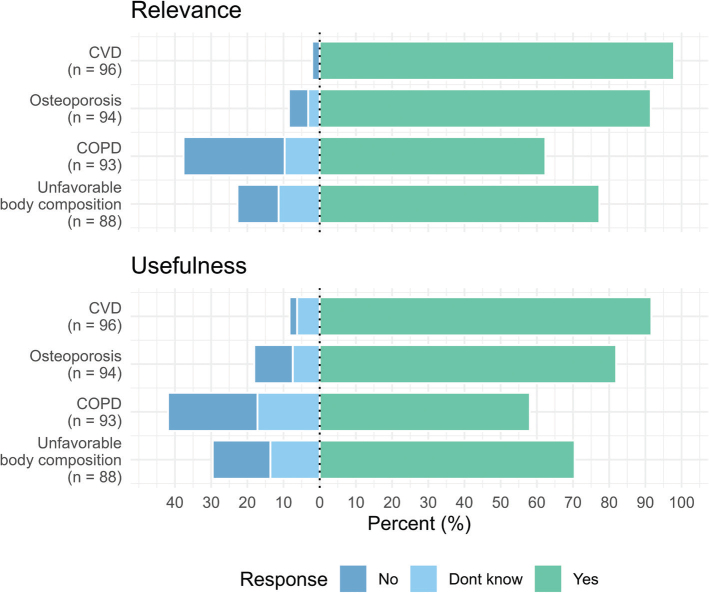
Perceived relevance and usefulness of AI-generated risk prediction of chronic diseases, shown in percentage. AI: artificial intelligence; CVD: cardiovascular disease; COPD: chronic obstructive pulmonary disease.

Responders expressed positive attitudes toward the relevance of AI-generated risk predictions across all four conditions. The highest perceived relevance was observed for CVD (97.9%, 95% confidence interval (CI) 92.7–99.7), followed by osteoporosis (91.5%, 95% CI 83.9–96.3), and unfavorable body composition (77.3%, 95% CI 67.1–85.5). Perceived relevance was lower for COPD (62.3%, 95% CI 51.7–72.2) (cf. Figure 1).

The pattern for perceived usefulness of AI-generated risk predictions was overall slightly lower. The highest perceived usefulness was observed for CVD (91.7%, 95% CI 84.2–96.3), followed by osteoporosis (81.9%, 95% CI 72.6–89.1), unfavorable body composition (70.5%, 95% CI 59.8–79.7), and COPD (58.1%, 95% CI 47.4–68.2).

In multivariate logistic regression analysis, increasing age was associated with higher odds of non-positive attitudes toward the usefulness of a COPD risk prediction model (Odds ratio [OR] 1.05 per year, *p* = 0.043) (Table 2). There was no evidence of an association between region and non-positive responses. Due to the small number of responders from suburban and rural institutions, location of institution was not included in the regression analyses.

**Table 2:** Multivariate logistic regression analysis.

Variable	*N*	Age aOR (95% CI)	Age *p*-value	Region *p*-value^a^
CVD relevant	96	Not analyzed^b^	-	-
CVD useful	96	Not analyzed^b^	-	-
Osteoporosis relevant	94	Not analyzed^b^	-	-
Osteoporosis useful	94	1.01 (0.96–1.07)	0.59	0.26
COPD relevant	93	1.02 (0.97–1.07)	0.45	0.62
COPD useful	93	1.05 (1.00–1.10)	0.043	0.81
Body composition relevant	88	1.01 (0.95–1.06)	0.18	0.35
Body composition useful	88	0.99 (0.94–1.04)	0.59	0.79

a Region *p*-value represent the overall effect of geographical region in the multivariate logistic regression model (likelihood ratio test)

b Multivariate logistic regression was not performed because fewer than 10 non-positive responses were observed. aOR: adjusted odds ratio; CI: confidence interval; CVD: cardiovascular disease; COPD: chronic obstructive pulmonary disease.

## Discussion and conclusion

This survey demonstrates that ROs overall are supportive of AI-generated risk predictions of chronic diseases using pCT-scans in eBC. Especially, the attitudes toward risk prediction for CVD and osteoporosis, both in terms of perceived relevance and usefulness, were positive. Although the majority reported positive attitudes toward risk predictions for COPD and unfavorable body composition, these predictions were perceived as less relevant and/or less useful.

The observed pattern suggests a clinically logical hierarchy. CVD and osteoporosis are well-established long-term complications of eBC treatment and are often integrated into survivorship care [2, 3]. An AI-generated risk prediction of these conditions may therefore be perceived as a natural extension of current clinical practice. In contrast, COPD and unfavorable body composition may be viewed as less directly attributable to eBC treatment, which may explain the lower proportion of positive responses for risk predictions of these conditions. Furthermore, low awareness of the association between unfavorable body composition and shorter time to recurrence could contribute to this tendency [10].

Increasing age was associated with less positive attitudes toward the usefulness of a COPD risk prediction model. No other associations between age and attitudes were found, suggesting that the acceptance toward AI-generated risk prediction may be consistent across age groups. No geographical differences were identified.

The study has limitations. The sample size was modest, resulting in too few non-positive responses for regression analysis in some disease categories. Additionally, the survey was online, and participation was voluntary, increasing the risk of self-selection and non-response bias. As a result, ROs with an interest in AI or survivorship may have been more likely to participate, potentially leading to overly positive findings. The study included only ESTRO-engaged ROs, who primarily originate from high-income countries. Furthermore, most responders were affiliated with urban institutions, resulting in poor representation from low-income and rural areas. Also, attitudes were assessed using a non-standardized three-category response scale, which will not capture nuanced views.

Nevertheless, this study provides an overall insight into ROs acceptance of AI-generated secondary risk prediction. While enthusiasm was high for CVD and osteoporosis, implementation of AI models for COPD and unfavorable body composition may require more clearly defined clinical pathways and stronger communication of their relevance and actionability. These findings parallel those of Eltorai et al., who reported that radiologists were generally receptive to AI-based opportunistic CT screening but expressed concerns regarding understanding of the models, perceived usefulness, and workflow integration [18]. Collectively, this evidence suggests that professional acceptance of AI-based secondary use of imaging depends not only on technological performance but also on evident clinical relevance, interpretability, and compatibility into existing workflows.

To our knowledge, this is the first study to specifically investigate ROs’ attitudes toward AI-based secondary utilization of pCT-scans in eBC survivorship care. While previous studies have explored radiotherapy professionals’ perceptions of AI applications such as image segmentation and dose optimization [19, 20], evidence regarding AI-based secondary risk prediction in radiotherapy remains limited. The generally positive attitudes observed in this study therefore address an important knowledge gap concerning the potential implementation of AI-generated risk prediction in survivorship care.

In conclusion, ROs expressed an overall high degree of support for AI-models to generate risk predictions for eBC patients, although enthusiasm varied by the individual disease. These findings indicate that clinical implementation is feasible, but disease-specific differences in perceived clinical value should be considered when integrating such models into practice.

## Data Availability

The data that support the findings of this study are available from the corresponding author upon reasonable request.
